# Noise Hazard Awareness Among Street Vendors and Evaluation of Noise Levels in the Market Areas of North Kolkata: A Cross-Sectional Study

**DOI:** 10.7759/cureus.104642

**Published:** 2026-03-04

**Authors:** Mohammed A Hussain, Rumelika Kumar, Narayani Siware, Kishore Eswaramoorthi, Ankur Chaudhari, Monalisha Sahu

**Affiliations:** 1 Preventive and Social Medicine, All India Institute of Hygiene and Public Health, Kolkata, IND; 2 Occupational Health, All India Institute of Hygiene and Public Health, Kolkata, IND

**Keywords:** noise-induced hearing loss, noise pollution, occupational exposure, occupational hazards, street vendors

## Abstract

Objectives: Prolonged exposure to high noise levels can result in noise-induced hearing loss (NIHL) and other adverse health outcomes. This study aimed to assess the awareness of noise hazards among street vendors in North Kolkata and to measure the noise levels in their work environment.

Methods: A descriptive cross-sectional study was conducted among 122 street vendors in the Hatibagan-Shyambazar market area between July and September 2023. Participants were selected using systematic random sampling from the market association list. A pretested, expert-validated structured questionnaire was used to assess awareness and self-reported auditory symptoms. Ambient noise levels were measured using a calibrated Class 1 sound level meter at three market locations (roadside stalls, inner lanes, and intersections) during peak business hours. Equivalent continuous sound level (Leq) values were compared with the Central Pollution Control Board (CPCB) permissible limits. Data were analyzed using SPSS Version 16 (SPSS Inc., Chicago, IL, USA). Logistic regression analysis was performed to identify factors associated with good awareness.

Results: Mean Leq levels exceeded CPCB limits (65 dB(A)) at all sites: roadside stalls (87.4 ± 4.5 dB(A)), inner lanes (82.1 ± 3.8 dB(A)), and intersections (89.8 ± 5.1 dB(A)). Although 82.8% of vendors were aware that loud noise can damage hearing, only 42.6% knew about government schemes for hearing impairment. Higher educational status was significantly associated with good awareness (aOR = 0.40; 95% CI: 0.17-0.94; p = 0.035).

Conclusion: Street vendors in North Kolkata are exposed to noise levels well above permissible limits, with notable gaps in awareness of noise-related hazards. Targeted awareness programs, provision of hearing protection, and strengthened regulatory enforcement are recommended.

## Introduction

Noise pollution is a well-established occupational hazard with significant implications for hearing and overall health. Prolonged exposure to high noise levels, particularly above permissible limits, is associated with noise-induced hearing loss (NIHL) and non-auditory effects, such as hypertension, sleep disturbances, and psychological stress [1,2]. Environmental noise has also been linked to cardiovascular morbidity [3]. The World Health Organization estimates that approximately 16% of disabling hearing loss in adults worldwide is attributable to occupational noise exposure [4].

Street vendors represent a substantial segment of the urban informal workforce and are routinely exposed to elevated noise from vehicular traffic, loudspeakers, public announcements, and market activities [5]. These workers largely operate outside formal occupational health regulations, resulting in prolonged and unmonitored exposure. Studies from commercial and market areas in India and other low- and middle-income countries have reported noise levels exceeding permissible limits, along with poor awareness of noise-related health risks [6-8]. However, many of these studies relied on self-reported exposure or lacked objective measurements using calibrated instruments.

Kolkata, one of India’s oldest and most densely populated metropolitan cities, is characterized by congested traffic and poorly planned market areas. Urban traffic noise in such settings has been associated with adverse cardiovascular outcomes, highlighting the public health relevance of occupational noise exposure among street vendors [3].

Although prior research has assessed environmental noise levels in urban settings and occupational exposure in formal industrial sectors, limited evidence exists regarding noise exposure among informal sector workers in urban marketplaces. In India, a substantial proportion of the urban workforce is employed in the informal sector, where occupational health safeguards are minimal or absent, making street vendors particularly vulnerable to chronic environmental hazards such as noise. Few studies have simultaneously measured ambient noise levels using calibrated instruments and evaluated awareness of noise-related health hazards among street vendors, and evidence from eastern India remains scarce. This lack of integrated assessments combining objective exposure measurements with awareness evaluation in informal marketplace settings represents an important knowledge gap. The present study aimed to assess awareness of noise hazards among street vendors in North Kolkata and to objectively measure ambient noise levels in their work environment using calibrated equipment and representative sampling.

## Materials and methods

Study setting and design

A descriptive cross-sectional study was conducted between July and September 2023 in the Hatibagan-Shyambazar market area of North Kolkata, West Bengal. This area is a major commercial hub, characterized by dense pedestrian traffic and heavy vehicular movement.

Study population and sampling

Street vendors aged 18 years and above who had been engaged in vending activities for at least one year were included in the study. Vendors who were acutely ill or unwilling to participate were excluded.

The sample size of 122 was calculated based on the prevalence of awareness reported in a previous study from Mysuru [6], using the formula n = Z²pq / d², where *Z* represents the standard normal deviate at 95% confidence level (1.96), *p* is the expected prevalence of adequate awareness regarding noise hazards as reported in the Mysuru study, *q* = 1 − p, and *d* is the allowable error, set at 10%. The sample size was calculated manually. After adjusting for a 20% non-response rate, the final estimated sample size was 120, and 122 participants were ultimately included. Systematic random sampling was performed using a vendor list obtained from the local market association.

Study tools and data collection

A pretested, content-validated structured questionnaire was used to collect data on sociodemographic characteristics, occupational history, awareness of noise hazards, self-reported auditory symptoms, and preventive practices. The questionnaire was content-validated by a panel of experts in community medicine and occupational health, with a content validity index (CVI) of 0.83, indicating good validity. Necessary modifications were incorporated prior to data collection. Data were recorded using the EpiCollect5 mobile platform (CGPS Team of Oxford BDI; https://five.epicollect.net), which allows real-time entry and GPS-based location tagging [9].

Ambient noise levels were measured using a Class 1 sound level meter compliant with IEC 61672-1 standards [10]. The instrument was calibrated before and after each measurement session using the built-in acoustic calibrator.

Noise measurement procedure

Noise assessments were conducted at roadside stalls, inner market lanes, and traffic intersections. Measurements were taken at a height of approximately 1.5 m using a tripod, with the microphone oriented toward the dominant noise source. Each measurement lasted 15 minutes and was performed during peak business hours (10:00-13:00 and 16:00-19:00) using A-weighting and slow response mode.

Equivalent continuous sound level (Leq), maximum sound level (Lmax), and minimum sound level (Lmin) were recorded. Measured values were compared with the Central Pollution Control Board (CPCB) permissible noise limit of 65 dB(A) for commercial areas [2].

**Figure 1:**
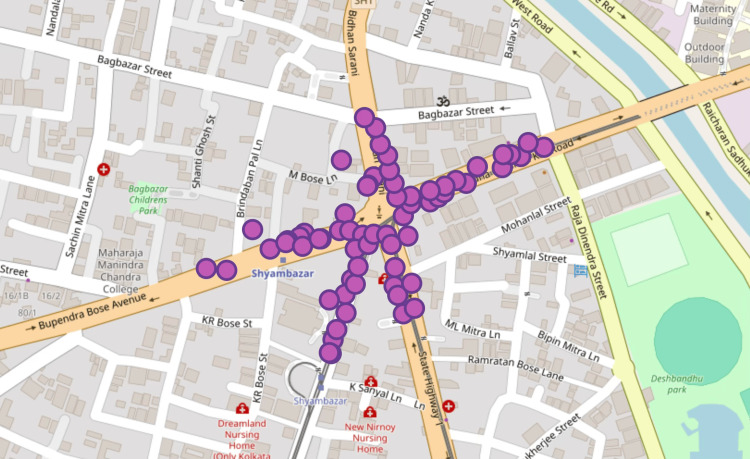
Geospatial distribution of noise measurement and survey sites in the Hatibagan-Shyambazar market area, North Kolkata.

GPS coordinates of survey and measurement locations were plotted on an OpenStreetMap base layer to illustrate the spatial distribution of data collection sites (Figure 1).

Data analysis

Data collected through EpiCollect5 were exported in CSV format and cleaned prior to analysis using SPSS Version 16 (SPSS Inc., Chicago, IL, USA) [11]. Descriptive statistics were used to summarize sociodemographic characteristics, awareness levels, and noise measurements. Chi-square or Fisher’s exact test was applied for categorical comparisons. Awareness was assessed using a structured questionnaire comprising multiple items on knowledge of noise hazards, health effects, and preventive practices. Each correct response was assigned a score of 1, while incorrect or “don’t know” responses were scored 0. A composite awareness score was calculated by summing individual item scores. Participants were categorized into “good awareness” and “poor awareness” groups based on the median score of the study population, with scores above the median classified as good awareness. Univariate and multivariate logistic regression analyses were performed to identify factors associated with good awareness. A p-value of less than 0.05 was considered statistically significant.

Ethical approval

Ethical approval for the analysis and publication of study data was obtained from the Institutional Ethics Committee (Approval No: IEC/2024(1)/51). The study was conducted in accordance with ethical principles, and written informed consent was obtained from all participants prior to data collection.

## Results

Sociodemographic profile

A total of 122 street vendors participated in the study. The mean age was 41.2 ± 9.6 years, with the largest proportion in the 40-49-year age group (31.1%). Males comprised 78.7% of the participants. Nearly one-third (34.4%) had completed secondary education, while 23.0% were illiterate. Approximately 34.4% of participants had been working in the market for more than 10 years (Table 1).

**Table 1:** Sociodemographic profile of the participants (N = 122).

Variable	Category	n (%)
Age group (years)	18-29	22 (18.0)
	30-39	35 (28.7)
	40-49	38 (31.1)
	≥50	27 (22.1)
Gender	Male	96 (78.7)
	Female	26 (21.3)
Education	Illiterate	28 (23.0)
	Primary	39 (32.0)
	Secondary	42 (34.4)
	Graduate and above	13 (10.6)
Years in market	1-5	41 (33.6)
	6-10	39 (32.0)
	>10	42 (34.4)

Ambient noise levels

The mean Leq exceeded the CPCB permissible limit of 65 dB(A) at all measured locations. Intersections recorded the highest mean Leq (89.8 ± 5.1 dB(A)), followed by roadside stalls (87.4 ± 4.5 dB(A)) and inner market lanes (82.1 ± 3.8 dB(A)). Overall, noise levels across all sites ranged from 75.5 to 97.5 dB(A) (Table 2).

**Table 2:** Measured noise levels at different market locations.

Location	Mean Leq (dB(A)) ± SD	Range
Roadside stalls	87.4 ± 4.5	79.8-94.2
Inner market lane	82.1 ± 3.8	75.5-88.9
Intersection area	89.8 ± 5.1	81.4-97.5
All sites combined	86.4 ± 5.0	75.5-97.5

Awareness regarding noise hazards

While 82.8% of vendors were aware that loud noise can damage hearing, only 65.6% knew that continuous noise exposure could lead to irreversible hearing loss. Awareness of government schemes for hearing impairment was low (42.6%). Nearly half of the respondents (49.2%) reported experiencing stress in noisy workplaces, and 28.7% reported tinnitus. Self-reported reduction in hearing sensitivity was noted in 36.1% of participants (Table 3).

**Table 3:** Awareness of noise hazards among participants.

Awareness question	Yes (%)	No (%)	Maybe (%)
Loud noise can damage hearing	101 (82.8)	9 (7.4)	12 (9.8)
Continuous noise causes irreversible hearing loss	80 (65.6)	19 (15.6)	23 (18.8)
Awareness of government schemes for hearing impairment	52 (42.6)	47 (38.5)	23 (18.9)
The necessity to protect the ears from noise	117 (95.9)	0 (0.0)	5 (4.1)
Experienced ringing/buzzing in ears (tinnitus)	35 (28.7)	63 (51.6)	24 (19.7)
Noticed hearing sensitivity reducing	44 (36.1)	58 (47.5)	20 (16.4)
Experienced stress in a noisy workplace	60 (49.2)	52 (42.6)	10 (8.2)

Factors associated with good awareness

On univariate analysis, vendors with secondary education or higher, age below 40 years, shorter duration of exposure (<10 years), and lower workplace noise levels (<85 dB(A)) had higher odds of good awareness compared to their respective reference categories. In multivariate logistic regression analysis, only education level remained independently associated with good awareness. Vendors with illiterate or primary-level education had significantly lower odds of good awareness compared to those with secondary education or higher (adjusted OR = 0.40; 95% CI: 0.17-0.94; p = 0.035) (Table 4). Reference categories were selected based on epidemiological relevance and the distribution of the study population. Odds ratios less than 1 indicate a lower likelihood of good awareness compared to the respective reference category.

**Table 4:** Univariate and multivariate logistic regression analysis of factors associated with good awareness of noise hazards (N = 122). Odds ratios less than 1 indicate lower odds of good awareness compared to the respective reference category. Reference categories were selected based on epidemiological relevance and distribution of the study population. *A p-value less than 0.05 was considered statistically significant.

Variable	Category	Unadjusted OR (95% CI)	p-value	Adjusted OR (95% CI)	p-value
Years of exposure	1-5 years	Ref	-	Ref	-
	6-10 years	0.64 (0.28-1.45)	0.284	0.71 (0.29-1.71)	0.446
	>10 years	0.39 (0.17-0.88)	0.024*	0.42 (0.17-1.04)	0.061
Age group	<40 years	Ref	-	Ref	-
	≥40 years	0.51 (0.26-0.98)	0.043*	0.59 (0.28-1.25)	0.169
Education level	Secondary and above	Ref	-	Ref	-
	Illiterate/primary	0.36 (0.16-0.79)	0.011*	0.40 (0.17-0.94)	0.035*
Daily working hours	≤8 hours	Ref	-	Ref	-
	>8 hours	0.56 (0.27-1.15)	0.115	0.63 (0.29-1.37)	0.243
Mean workplace noise level	<85 dB(A)	Ref	-	Ref	-
	≥85 dB(A)	0.42 (0.20-0.89)	0.023*	0.48 (0.21-1.08)	0.075
History of tinnitus	No	Ref	-	Ref	-
	Yes	1.90 (0.83-4.36)	0.128	1.76 (0.74-4.21)	0.202
Self-reported hearing difficulty	No	Ref	-	Ref	-
	Yes	2.37 (0.96-5.84)	0.061	2.11 (0.82-5.43)	0.122

## Discussion

Street vendors in North Kolkata were exposed to ambient noise levels well above recommended limits. Intersections recorded the highest levels, consistent with other Indian market-based studies that identify traffic junctions as dominant noise sources [7,8]. Prolonged exposure at these levels poses a substantial risk for NIHL, as well as non-auditory outcomes, including cardiovascular and psychological effects [1,3].

Although environmental and occupational noise exposure has been examined in industrial and traffic-dense urban settings, evidence from informal urban marketplaces remains limited. Few studies have combined objective ambient noise measurements using calibrated instruments with assessments of awareness regarding noise-related health hazards among informal sector workers. This study adds context-specific evidence from an eastern Indian metropolitan market and highlights the occupational health vulnerability of street vendors, who largely operate outside formal occupational health surveillance and regulatory frameworks [6,12].

The mean Leq observed in this study, ranging from 75.5 to 97.5 dB(A), is comparable to findings from other commercial and marketplace settings in low- and middle-income countries. Studies from Indian commercial areas and informal urban markets in Nigeria have documented ambient noise levels between 80 and 95 dB(A), frequently exceeding permissible limits and posing a risk for occupational hearing loss [7,8,13,14]. Similar to our findings, these studies also reported limited use of hearing protection devices and gaps in awareness regarding the irreversible nature of noise-induced hearing impairment among informal workers [6,14].

Although most vendors recognized that loud noise could damage hearing, awareness regarding the irreversible nature of NIHL and available preventive or rehabilitative services was lower. Similar gaps have been reported among informal sector workers in other low- and middle-income country settings, including marketplace and small-scale industrial environments in India and Nigeria, where elevated ambient noise levels and low adoption of hearing protection have been documented [6,15]. Informal occupations remain particularly vulnerable due to limited occupational health surveillance, poor access to preventive services, and minimal use of hearing protection devices [12].

Educational status emerged as a significant determinant of awareness, consistent with evidence that higher education is associated with improved understanding of occupational health risks [15]. Duration of exposure and measured noise levels did not retain statistical significance after adjustment, suggesting that knowledge gaps may persist irrespective of exposure intensity and highlighting the need for targeted awareness interventions tailored to low-literacy occupational groups.

Measured noise levels consistently exceeded the 85 dB(A) threshold associated with an increased risk of occupational hearing loss [1]. Despite a high perceived risk and reported auditory symptoms, none of the vendors reported regular use of hearing protection, a finding consistent with studies among informal workers in other low- and middle-income countries [14]. The combination of elevated noise exposure and inadequate preventive practices across diverse informal market environments underscores the persistent and under-recognized occupational health risks faced by street vendors, reinforcing the public health relevance of localized assessments.

Strengths and limitations

This study employed a calibrated Class 1 sound level meter, GPS-based spatial mapping, and systematic sampling from a vendor registry. Limitations include reliance on self-reported data, the cross-sectional design, and the restriction of noise measurements to peak business hours. Due to the study design and fixed-location measurements, reproducibility and causal relationships could not be established, and cumulative noise exposure or individual dose-response relationships could not be evaluated in the absence of personal dosimetry or long-term monitoring.

## Conclusions

Street vendors in North Kolkata are exposed to ambient noise levels that consistently exceed permissible limits for commercial areas. While many participants were generally aware of the harmful effects of loud noise, significant gaps were noted in knowledge regarding the irreversible nature of NIHL and the use of preventive measures. These findings underscore the need for targeted awareness programs, improved access to basic hearing protection, and the inclusion of informal sector workers in urban noise monitoring and occupational health planning.

## Appendices

Standard operating procedure (SOP) for measurement of ambient noise levels in market areas

Purpose

To standardize the procedure for measuring ambient noise levels, expressed as equivalent continuous sound level (Leq) in dB(A), in market environments.

Equipment

  Class 1 sound level meter (Cygnet Acoustic Calibrator™)

  Tripod stand

  Field notebook or digital recording device

  GPS-enabled device

Procedure

Calibration: The sound level meter is calibrated before and after each measurement session using the built-in acoustic calibrator.

Site selection: Measurement sites are identified across different market locations, including roadside stalls, inner market lanes, and traffic intersections. GPS coordinates of each site are recorded.

Measurement setup: The sound level meter is mounted on a tripod at a height of approximately 1.5 m above ground level, with the microphone oriented toward the predominant noise source.

Measurement duration and settings: Noise measurements are conducted for a duration of 15 minutes at each site during peak business hours. A-weighting and slow response mode are used for all recordings.

Data recording: Equivalent continuous sound level (Leq), maximum sound level (Lmax), and minimum sound level (Lmin) are recorded. Observational notes regarding traffic density, public announcements, and surrounding activities are documented.

Data handling and interpretation: Recorded data are stored securely and backed up. Measured noise levels are compared against the Central Pollution Control Board (CPCB) permissible limits for commercial areas.

Safety considerations

Measurements are conducted without obstructing pedestrian movement or vendor activities. Field investigators carry institutional identification during data collection.

This SOP was developed in accordance with the IEC 61672-1 standards and Central Pollution Control Board (CPCB) noise-monitoring guidelines.
